# Oseltamivir use and outcomes during the 2009 influenza A H1N1 pandemic in Taiwan

**DOI:** 10.1186/1471-2458-13-646

**Published:** 2013-07-12

**Authors:** Chia-Hung Liu, Jiun-Ling Wang, Chia-Ping Su, Jen-Hsiang Chuang, Chia-Hsuin Chang, Mei-Shu Lai

**Affiliations:** 1 Institute of Epidemiology and Preventive Medicine, College of Public Health, National Taiwan University, Taipei, Taiwan; 2 Department of Family Medicine, Taipei Medical University-Shuang Ho Hospital, New Taipei, Taiwan; 3 Department of Internal Medicine, E-Da hospital/ I-Shou University, Kaohsiung, Taiwan; 4 Taiwan Centers for Disease Control, Taipei, Taiwan; 5 Department of Internal Medicine, National Taiwan University Hospital, Taipei, Taiwan

**Keywords:** Oseltamivir, Influenza A virus, H1N1 subtype, Pandemic, Mortality

## Abstract

**Background:**

The Taiwan CDC provided free oseltamivir to all patients with influenza infections confirmed by rapid testing or who had clinical warning symptoms during the 2009 H1N1 influenza pandemic in Taiwan. However, oseltamivir utilization patterns, cost, and outcomes among oseltamivir-treated patients remained unclear.

**Method:**

A population-level, observational cohort study was conducted using the Taiwan National Health Insurance Database from January to December 2009 to describe the use of oseltamivir.

**Result:**

Prescription trend over weeks increased after a change in government policy and responded to the influenza virus activity. The overall prescription rate was 22.33 per 1000 persons, with the highest prescription rate of 116.5 for those aged 7–12 years, followed by 69.0 for those aged 13–18 years, while the lowest rate was 1.7 for those aged ≥ 65 years. As influenza virus activity increased, the number of prescriptions for those aged ≤18 years rose significantly, whereas no substantial change was observed for those aged ≥65 years. There were also regional variations in terms of oseltamivir utilization and influenza complication rates.

**Conclusions:**

Oseltamivir was widely used in the 2009 H1N1 influenza pandemic in Taiwan, particularly in those aged 7–18 years. The number of prescriptions for oseltamivir increased with a change in government policy and with increasing cases of pandemic influenza. Further study is needed to examine whether there is an over- or under-use of anti-influenza drugs in different age groups or regions and to examine the current policy of public use of anti-influenza drugs to reduce influenza-associated morbidity and mortality.

## Background

The 2009 influenza A H1N1 pandemic was responsible for at least 18,000 deaths worldwide from March 2009 throughout August 2010 [1]. Oseltamivir, a neuraminidase inhibitor, is a widely-used anti-influenza agent to treat and prevent seasonal or pandemic influenza infections. The World Health Organization (WHO), the US Centers of Disease and Control (CDC), and the European Centre for Disease Prevention and Control recommend that governments stockpile and properly deliver anti-influenza agents to diminish the impact of pandemic influenza [2-4].

Despite the reports that oseltamivir may reduce influenza complications and hospitalizations, recent Cochrane reviews have found that oseltamivir can mitigate influenza-like illness (ILI) symptoms for 21 hours. However, the authors are unable to conclude the effect of oseltamivir on complications or transmission [5]. Based on the current evidence, criticisms have been raised of the public policy calling for mass oseltamivir prescription during the 2009 influenza A H1N1 pandemic [6].

In Taiwan, the first incidence of H1N1 infection was reported on May 20, 2009 and the first death occurred on July 30, 2009. To control viral transmission and reduce influenza-related morbidity and mortality, the Taiwan Centers of Disease Control (Taiwan CDC) has provided free antiviral agents (oseltamivir and zanamivir) since August 15, 2009 through the Bureau of National Health Insurance (BNHI) to all patients with influenza infections that were confirmed by rapid diagnostic testing, or to those with influenza danger signs defined by WHO (including symptoms and signs suggesting oxygen impairment, cardiopulmonary insufficiency or central nervous system complications such as shortness of breath, tachypnea, altered mental status etc.) during the pandemic [3]. Receiving prescriptions for prophylaxis or stockpiling purposes was not allowed in the national health insurance program. However, under this policy, the utilization patterns, cost, and outcomes among oseltamivir-treated patients remained unclear. A clear understanding of oseltamivir utilization is important in terms of national medical resource allocation and policy making for disease control. Meanwhile, examining the utilization patterns of anti-influenza use can reveal whether clinicians have followed guidelines to protect high risk patient groups [7]. Therefore, we conducted a population-level, observational cohort study using the BNHI database from January to December 2009 to describe the frequency of oseltamivir use and outcomes of influenza patients in Taiwan.

## Methods

### Ethics

Taiwan Centers for Disease Control Research approved the protocol of this study and waived the need for written informed consent because this is a retrospective study based on data from administrative databases and involved only minimal risk.

### Data source

In 1995, a compulsory, single-payer national health insurance (NHI) program was implemented in Taiwan. The NHI claims database includes complete records of outpatient visits, hospital admissions, prescriptions, disease states and vital status for 99% of the 23 million Taiwanese inhabitants. Influenza surveillance systems in Taiwan have been described in another paper [8]. We established longitudinal medical histories for each beneficiary by linking several computerized administrative and claims datasets, and National Death Registry data through the civil identification number unique to each beneficiary and the date of birth.

### Study population

We identified patients who received oseltamivir prescriptions (anatomical therapeutic chemical [ATC] classification system code J05AH02) between January 1 and December 31, 2009. Data were further stratified according to prescribers’ characteristics (outpatient or inpatient use, region, and medical facility).We collected information of prescribed drug supply days, date of prescription, and total number of pills dispensed from the pharmacy prescription database. Data on patient characteristics, including age, gender, influenza diagnosis and related complications were obtained from inpatient and outpatient medical records.

### Statistical analysis

The primary variables included the number of oseltamivir prescriptions and the rate of prescription per 1,000 persons in the overall population and for each age group, calculated with the use of 2009 census statistics in Taiwan. Prescription trend over weeks was reported in relation to influenza virus activity, as measured by positive H1N1 virus isolation rate from the Taiwan Centers for Disease Control Influenza Surveillance Virology Laboratory [8]. We also estimated the expenditures for oseltamivir and the proportion relative to the total outpatient expenditures related to influenza treatment.

To investigate the outcomes of oseltamivir-treated patients, we reported the hospitalization rate of patients who received oseltamivir treatment during outpatient visits and the proportion of patients hospitalized for influenza and related complications leading to a poor outcomes, which was defined as respiratory failure or death. We also calculated the influenza complication rates in different age groups and regions based on the influenza surveillance data from Taiwan CDC.

## Results

### Utilization pattern

**Figure 1:**
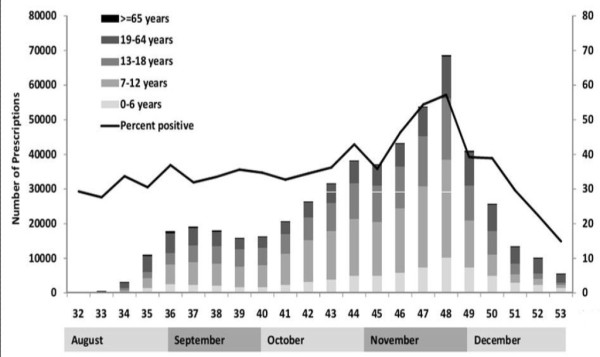
Weekly oseltamivir outpatient prescriptions across different age groups from August to December 2009 in Taiwan and percentage of positive influenza surveillance.

**Figure 2:**
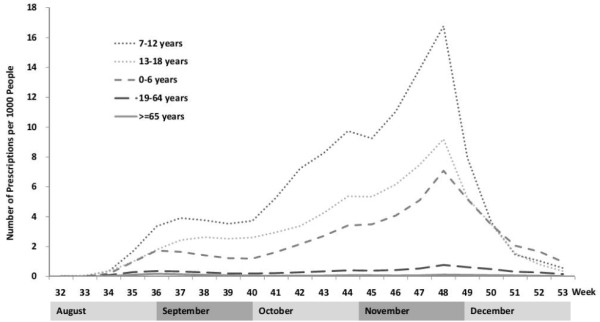
Number of prescriptions across different age groups and rate of prescription per 1000 persons across different age groups.

A total of 516,772 oseltamivir outpatient prescriptions were identified in 2009 in Taiwan. In contrast to only 489 prescriptions during January and July, the number of oseltamivir prescriptions increased dramatically after the oseltamivir reimbursement policy began in August, reaching its peak at week 48 and then gradually decreasing after the mass vaccination program began in November. The prescription trend was in general agreement with the H1N1 influenza virus positive isolation rate (Figure 1). Among the positive specimens in 2009 pandemic, there were 79.7% of influenza A (H1N1). Most oseltamivir use was for those aged 7–12 years (38.0%), whereas only 0.8% were prescribed for those aged ≥65 years (Table 1). The overall prescription rate was 22.33 per 1000 persons. Oseltamivir prescription trends varied for different age groups (Figure 2). Among all age groups, the highest prescription rate per 1000 persons was 116.5 for those aged 7–12 years, followed by 69.0 for those aged 13–18 years, while the lowest rate was 1.7 for those aged ≥ 65 years. The number of prescriptions for those aged ≤18 years rose significantly as influenza virus activity increased, whereas no substantial change was observed for those aged ≥65 years (Figure 2).

**Table 1:** Total number and rate of oseltamivir prescription and complication rates from August to December 2009 in Taiwan

	**Number of prescriptions (%) (N = 516,283)**	**Prescription rate per 1000 persons**	**Influenza with complications per million persons**
Age group			
0-6	74,432 (14.4)	51.4	139.4
7-12	196,086 (38.0)	116.5	74.5
13-18	133,153 (25.8)	69.0	65.2
19-64	108,473 (21.0)	7.0	28.8
≥65	4,139 (0.8)	1.7	54.1
Region			
A	187,655 (36.4)	25.2	64.2
B	111,249 (21.6)	32.1	47.9
C	98,109 (19.0)	21.9	27.5
D	53,929 (10.4)	15.8	28.7
E	53,640 (10.4)	14.3	34.7
F	11,646 (2.3)	20·3	172.6

Note: Region A and B were in northern Taiwan. Region C was in central Taiwan. Region D and E were in southern Taiwan. Region F was in eastern Taiwan. Influenza with complications: influenza danger signs defined by WHO (including symptoms and signs suggesting oxygen impairment, cardiopulmonary insufficiency or central nervous system complications such as shortness of breath, tachypnea, altered mental status etc.).

**Figure 3:**
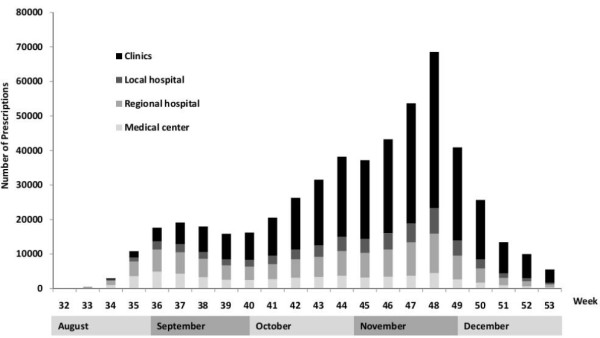
Number of prescriptions from different medical facilities.

There was a substantial difference in oseltamivir prescription rates among different regions, with higher utilization in northern Taiwan than in southern Taiwan (Table 1). Overall, 57.7% of oseltamivir was prescribed by primary care doctors, followed by 20.5% by regional hospitals, and 10.9% for local hospitals and medical centers (Figure 3). Of note, oseltamivir was mostly prescribed by physicians in regional hospitals and medical centers in August, whereas prescriptions by primary care doctors substantially increased after September (Figure 3).

A total of 487,278 patients received oseltamivir treatment during outpatient visits and 1,535 patients received oseltamivir therapy while hospitalized from August and December 2009. As compared to patients receiving outpatient treatment, a greater proportion of hospitalized patients received oseltamivir treatment in the age groups 0–6 and ≥65 years (Table 2).

**Table 2:** Patients (%) receiving outpatient and inpatient oseltamivir treatment in 2009

	**Outpatient treatment (%) (N = 487,278)**	**Inpatient treatment (%) (N = 1,535)**
Age group		
0-6	14.2	27.4
7-12	38.2	28.7
13-18	26.1	13.0
19-64	20.7	23.2
≥65	0.8	7.6
		
Hospital levels		
Primary care doctor	57.7	−
Local hospitals	10.9	18.8
Regional hospitals	20.5	56.9
Medical centers	10.9	24.2

In 2009, expenditures for oseltamivir were 14.2 millions US dollar (60.9 US dollar per 1,000 persons), accounting for 37.2% of the total expenditures related to outpatient visits for oseltamivir users.

### Outcomes of pandemic influenza A H1N1 and related complications

Among the oseltamivir-treated patients, 11.2% were diagnosed with a lower respiratory tract infection or pneumonia. Approximately 2.0% of patients received outpatient oseltamivir treatment requiring hospitalization, whereas 2.7% of hospitalized patients incurred respiratory failure or death. Among patients with influenza complications, 18.5% were aged < 6 years, 69.4% were aged 6–65 years, and 12.2% were aged > 65 years. Thirty-two percent (n=305) had underlying diseases. Most common underlying diseases reported were chronic lung disease (n=73), metabolic diseases (n=71), and cardiovascular diseases (n=54). The complication rates in different age groups and regions were also shown in Table 1. Region D and E, which had the lowest oseltamivir prescription rates among all regions, did not have the highest complication nor mortality rates.

## Discussion

Our study described the prescription pattern of oseltamivir in Taiwan during the 2009 influenza A H1N1 pandemic. Oseltamivir was widely used in Taiwan, with an overall prescription rate of 22.33 per 1000 persons. Despite the majority of outpatient use in school-aged children and adolescents, a substantially higher proportion of hospitalized patients receiving oseltamivir were those aged 0–6 years and 65 years or older. Our research did not show higher utilization of anti-influenza drugs associated with better outcome in population level during influenza pandemic. The benefits of anti-influenza therapy may vary among different age groups and geographical areas.

During the 2009 influenza A H1N1 pandemic, the US CDC and the WHO suggested prompt empirical neuraminidase inhibitor treatment for those with complicated illnesses regardless of previous health status and those at high risk for severe disease, such as children younger than 2 years old, adults aged 65 years and older, pregnant women, and those with chronic heart, lung, liver, kidney disorders, and weakened immune systems [4,9]. It was also recommended that treatment should start as early as possible even before definitive diagnostic test results become available [4,9]. In Taiwan, the government provided free anti-influenza treatment through the NHI system for influenza patients with either positive rapid diagnostic test results or with WHO defined danger signs [3]. And this free anti-influenza treatment policy did influence the usage pattern of anti-influenza drugs in Taiwan. The first peak of virus isolation of H1N1 in 35–36 week did not coincide with the usage of oseltamivir (data not shown). This may be explained by the Taiwan CDC providing free antiviral agents since August 15, 2009. Despite a concern that oseltamivir overuse could select resistant viruses, studies that examined countries with high levels of oseltamivir use did not find a significant percentage of oseltamivir resistant influenza virus emerging during H1N1 pandemic [10-12].

Our study showed that there was a substantial difference in oseltamivir use among age groups. The highest outpatient prescription proportion was observed for those aged 7–18 years old (64.3%), which was probably due to the high infectious rate in this age group [13-15]. However, according to current treatment guidelines, patients in this age group, who have uncomplicated illness and are not in a group known to be at higher risk of developing severe or complicated illness, may not need to be treated with neuraminidase inhibitors [2-4]. In contrast, individuals aged 65 years or older had a much lower outpatient prescription rate (0.8%) despite that they were more vulnerable to severe influenza-related complications, if they were to acquire an infection. From the ICD-9 diagnosis in outpatients department visit in Taiwan in 2009 pandemic, children and adolescents had about four-fold higher numbers of ILI visit comparison to those aged 65 years or older [7]. Children and adolescents had a six-fold higher incidence of seropositivity in comparison to those aged 65 years or older [13]. However, children and adolescents (aged 7–18) had a more than fifty-fold higher numbers of the anti-influenza prescriptions in comparison to those aged 65 years or older in this study. Similar findings were observed in the US; clinicians were less likely to provide anti-influenza agents to adults ≥65 years old with influenza, compared with younger adults [15]. The low oseltamivir utilization in elderly may be explained by atypical presentation in this age group and/or decreased sensitivity of rapid influenza diagnostic testing [16-18]. The use of rapid diagnostic tests would ensure oseltamivir were only prescribed to people with confirmed influenza, reducing inappropriate use [18]. However, reliance on typical influenza symptoms and rapid influenza diagnostic tests for clinical decision-making may lead to many influenza infections going untreated or causing a treatment delay [16]. A global pooled analysis showed the relative risk of death was highest in the age groups 50–64 and ≥65 years and the presence of chronic illness increased the likelihood of death [17]. In Taiwan, the elderly had a three-fold higher incidence of serious infection compared to adolescents [13]. Further research is needed to evaluate whether there is an over- or under-utilization of anti-influenza agents among different age groups.

**Figure 4:**
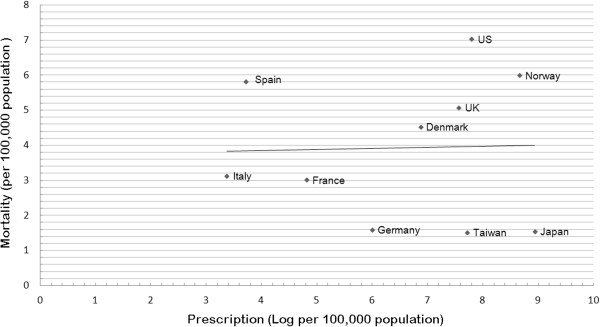
**Oseltamivir prescription rates and mortality of influenza patients among different countries.** X axis: prescription (Log per 100,000 population); Y axis :mortality (per 100,000 population).

From a literature search from Pubmed, Medline and Google using keyword “oseltamivir”, “prescription” and country names, we identified 10 published articles and governmental reports on oseltamivir prescriptions in 2009 H1N1 pandemic (Table 3) (Figure 4) [19-24]. We collected the reported number of cumulative confirmed fatal 2009 pandemic influenza A (H1N1) from European Centre for Disease Prevention and Control [25]. A substantial difference in oseltamivir prescription rates was found among countries, with the highest rates in Japan and Norway. There was no significant relation between prescription rate and influenza-associated mortality at the population level by linear regression (slope of the regression line 0.03; p=0.933). This may be explained by different prescription policy and accessibility of anti-influenza drugs during H1N1 pandemic in different countries. Healthcare accessibility and the lack of a co-payment for anti-influenza therapy made Taiwan become one of the countries with the highest oseltamivir utilization in the world. We did not know whether high mortality even under high oseltamivir prescription was related to (1) most prescriptions were probably issued to those people with mild cases of influenza (2) delayed oseltamivir use; (3) poor prognosis in certain risk groups; (4) bacterial co-infections [26] or (5) different mortality definition. Some physical and social factors may impede access to early use of anti-influenza drugs with the potential for more serious influenza outcomes. Besides, our data also showed regional differences in the oseltamivir utilization and influenza complication rate. In fact, the oseltamivir utilization rate was higher in northern Taiwan than southern Taiwan, and the complication rate was highest in eastern Taiwan. There are many factors that can influence the regional variations in terms of oseltamivir utilization rates and influenza complication rates. For example, influenza virus activity and number of infected cases, demographics and clinical risk profiles of the population, time to access to the healthcare system, physician attitude on anti-influenza prescription, and the quality of outpatient and inpatient care, may all play a role. From these data, it is difficult to make any conclusion about the potential relation between oseltamivir use and influenza outcomes. However, the observed excess variability may suggest a disparity in terms of resource allocation among different regions in Taiwan. Public health efforts to overcome social factors to provide early anti-influenza drugs access may decrease influenza-related mortality [9]. Further study investigating optimal public anti-influenza drugs delivery and use, and research identifying area of inefficiency to correct potential under- or over-utilization is urgently needed to create strategies to prepare for the coming influenza pandemics.

**Table 3:** Oseltamivir prescription and mortality rate in country level during 2009 H1N1 pandemic from literature review

**Country**	**Reference**	**Population (millions)**	**Study period**	**Oseltamivir per 100,000 population**	**Mortality rate per million (Ref 8)**
Japan	Ref 9	128	Aug 2009- Mar 2010	7625	1.55
Norway	Ref 10	4.8	Jan 2009- Dec 2009	5795.6	6.00
US	Ref 11	307	Apr 2009- Mar 2010	2420.5	7.04
Taiwan	This article	23.1	Aug 2009-Dec 2009	2233.1	1.52
UK	Ref 12	60.6	July 2009- Feb 2010	1933.6	5.08
Denmark	Ref 13	5.5	Jan 2009- Dec 2009	959.6	4.53
Germany	Ref 14	82.6	Oct 2009- Dec 2009	405.3	1.6
France	Ref 14	65	Oct 2009- Dec 2009	122.6	3.02
Spain	Ref 14	46.6	Oct 2009- Dec 2009	40.9	5.82
Italy	Ref 14	60	Oct 2009- Dec 2009	29.3	3.13

There were several limitations in our study. First, the database did not include self-administered oseltamivir use. As there was a supply shortage of oseltamivir during the 2009 H1N1 pandemic, oseltamivir prescription data in the NHI database could represent the use of anti-influenza drugs throughout the entire country. Second, because the accuracy of influenza infection based on ICD-9-CM diagnostic codes was low, we only examined the infection outcomes among those who received oseltamivir treatment. Third, due to a lack of related information, we only examined the relation between crude oseltamivir utilization rate and crude influenza-related mortality without taking into account age distribution.

## Conclusion

In conclusion, our study found that oseltamivir was widely used during the 2009 H1N1 pandemic in Taiwan, particularly those aged 7–18 years. Further study is needed to examine whether there is an over- or under-use of anti-influenza drugs in different age groups or regions and to examine the current policy of public use of anti-influenza drugs reduce influenza-associated morbidity and mortality.

## Competing interests

The authors declare that they have no competing interests.

## Authors’ contributions

The study was jointly designed by all authors. CHL and CPS were responsible for the data analyses. MSL was the PI of the cohort study and main responsible for data acquisition. CHL, JLW and CHC wrote the first draft. CPS and JHC contributed to subsequent drafts of the paper. All authors contributed to successive drafts. All authors read and approved the final manuscript.

## Pre-publication history

The pre-publication history for this paper can be accessed here:

http://www.biomedcentral.com/1471-2458/13/646/prepub
